# Leptin/obR signaling exacerbates obesity-related neutrophilic airway inflammation through inflammatory M1 macrophages

**DOI:** 10.1186/s10020-023-00702-w

**Published:** 2023-07-24

**Authors:** Yang Wang, Rongjun Wan, Chengping Hu

**Affiliations:** 1 Department of Respiratory Medicine (Department of Respiratory and Critical Care Medicine), Xiangya Hospital, Central South University, Changsha, 410008 Hunan People’s Republic of China; 2 Department of Geriatrics, The Second Xiangya Hospital, Central South University, Changsha, 410008 Hunan People’s Republic of China

**Keywords:** Obesity, Neutrophilic airway inflammation, Leptin, Leptin receptor, M1 macrophage polarization

## Abstract

**Background:**

Obesity-related asthma is a kind of nonallergic asthma with excessive neutrophil infiltration in the airways. However, the underlying mechanisms have been poorly elucidated. Among the adipokines related to obesity, leptin is related to the inflammatory response. However, little is understood about how leptin acts on the leptin receptor (obR) in neutrophilic airway inflammation in obesity-associated asthma. We explored the inflammatory effects of leptin/obR signaling in an obesity-related neutrophilic airway inflammation mouse model.

**Methods:**

We established a neutrophilic airway inflammation mouse model using lipopolysaccharide (LPS)/ovalbumin (OVA) sensitization and OVA challenge (LPS + OVA/OVA) in lean, obese, or db/db (obR deficiency) female mice. Histopathological, bronchoalveolar lavage fluid (BALF) inflammatory cell, and lung inflammatory cytokine analyses were used to analyze airway inflammation severity. Western blotting, flow cytometry, reverse transcription‐polymerase chain reaction (RT-PCR), and enzyme-linked immunosorbent assay (ELISA) were used to evaluate the underlying mechanisms. In vitro bone marrow‐derived macrophage (BMDM) and bone marrow-derived neutrophil experiments were performed.

**Results:**

We found that the serum leptin level was higher in obese than in lean female mice. Compared to LPS/OVA + OVA-treated lean female mice, LPS/OVA + OVA-treated obese female mice had higher peribronchial inflammation levels, neutrophil counts, Th1/Th17-related inflammatory cytokine levels, M1 macrophage polarization levels, and long isoform obR activation, which could be decreased by the obR blockade (Allo-Aca) or obR deficiency, suggesting a critical role of leptin/obR signaling in the pathogenesis of obesity-related neutrophilic airway inflammation in female mice. In in vitro experiments, leptin synergized with LPS/IFN-γ to promote the phosphorylation of the long isoform obR and JNK/STAT3/AKT signaling pathway members to increase M1 macrophage polarization, which was reversed by Allo-Aca. Moreover, leptin/obR-mediated M1 macrophage activity significantly elevated CXCL2 production and neutrophil recruitment by regulating the JNK/STAT3/AKT pathways. In clinical studies, obese patients with asthma had higher serum leptin levels and M1 macrophage polarization levels in induced sputum than non-obese patients with asthma. Serum leptin levels were positively correlated with M1 macrophage polarization levels in patients with asthma.

**Conclusions:**

Our results demonstrate leptin/obR signaling plays an important role in the pathogenesis of obesity-related neutrophilic airway inflammation in females by promoting M1 macrophage polarization.

**Graphical abstract:**

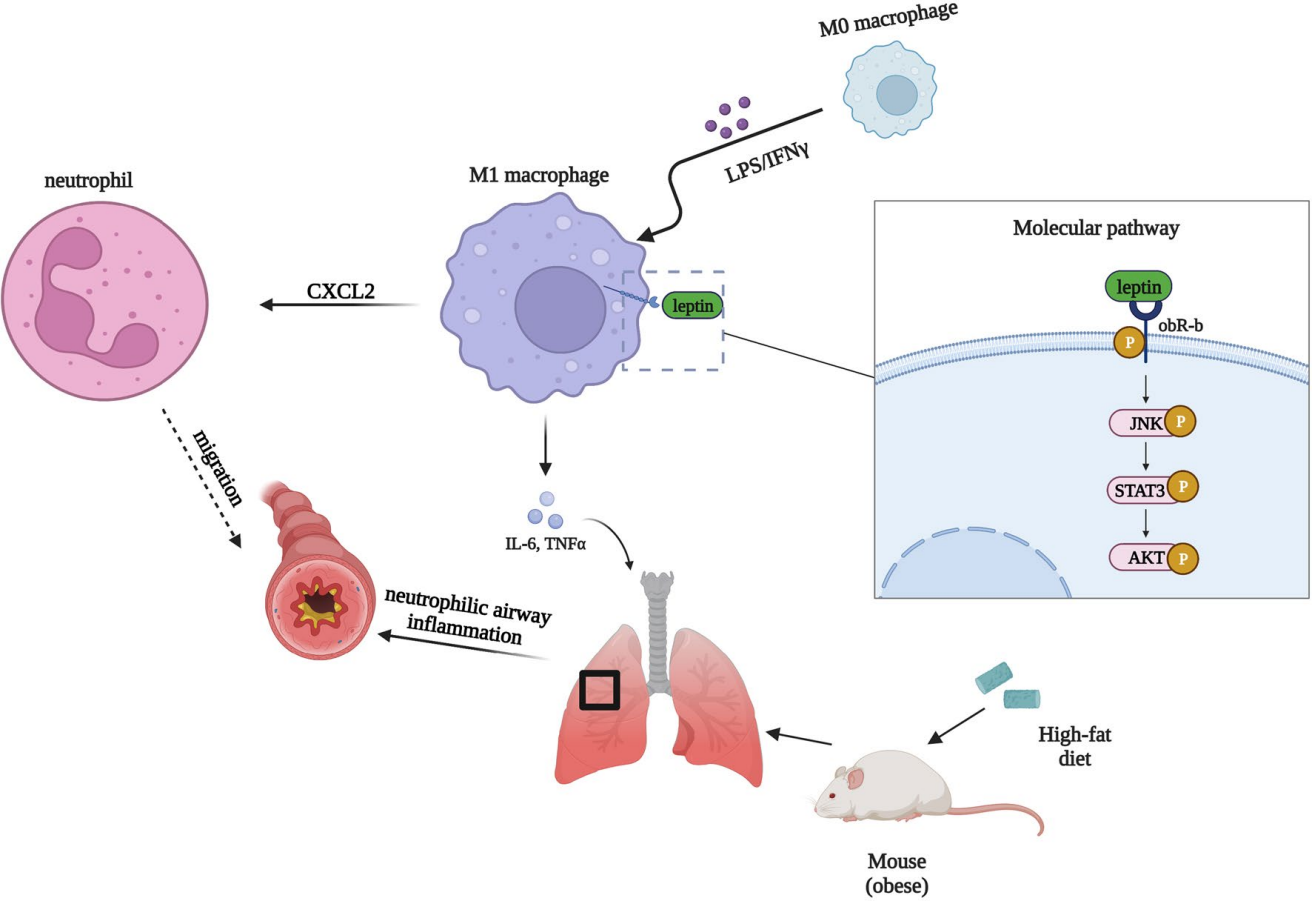

**Supplementary Information:**

The online version contains supplementary material available at 10.1186/s10020-023-00702-w.

## Background

Asthma is a respiratory disease characterized by chronic and heterogeneous airway inflammation, airway hyper-responsiveness, and airflow restriction, the pathogenesis of which is closely related to T helper 1 (Th1)/T helper 2 (Th2) immune response imbalance (Kim et al. 2017a; Ye et al. 2017; Wenzel 2012). The Th1 immune response includes a specific cytokine secretion profile and immune functions and is defined by IL-12 and IFN-γ production and cell-mediated responses (Macatonia et al. 1993, 1950), while the Th2 immune response is defined by the production of IL-4, IL-10, and antibody responses (Romagnani 1994). Th2 cells play vital roles in eosinophilic asthma (Robinson et al. 1992), while Th1 cells play an important role in neutrophilic asthma (Newcomb and Peebles 2013). The incidence of obesity is increasing worldwide, leading to a series of social problems, such as productivity loss, a high rate of chronic diseases, and increased use of sick days (Valsamakis et al. 2017). Obesity has been reported to be a prevalent risk factor for asthma severity (Wenzel 2012; Kim et al. 2014). Obesity-related asthma is a type of nonallergic asthma involving increased levels of airway neutrophils and few levels of airway eosinophils, which can lead to a poor response to conventional therapies (Peters et al. 2018; Thompson et al. 2021; Chen et al. 2006). However, the underlying mechanisms of obesity-related asthma pathophysiology are not completely understood. It was reported that the prominent features of obesity-related asthma were increased systemic/airway inflammation and severe symptoms (Liu et al. 2018). Weight loss can reduce the level of proinflammatory molecules and increase the level of anti-inflammatory molecules (Baltieri et al. 2018). However, it is difficult for most obese patients to change their weight within a short time. Even after a low-calorie diet, only 25% of obese patients could maintain long-term weight control (Flore et al. 2022). Therefore, therapies for obesity-related neutrophilic airway inflammation will need to not only control weight but also target proinflammatory molecules. Compared with nonobese asthmatic patients, asthmatic patients with obesity showed increased neutrophilic airway inflammation and increased levels of inflammatory chemicals derived from adipocytes (Moore et al. 2014; Scott et al. 2011). However, the mechanisms by which pathogenic neutrophils infiltrate into the obesity-related airway in the context of obesity-associated asthma remain largely unclear.

Leptin, encoded by an obesity-related gene, is mainly secreted by adipocytes (Chan et al. 2003; Jutant et al. 2021). Circulating levels of leptin are proportional to body fat mass (Chrysafi et al. 2020). Leptin was previously studied in immune and inflammatory responses (Cava and Matarese 2004; Abella et al. 2017). Binding of leptin to its receptor (leptin receptor, obR) could contribute to downstream intracellular signaling activation (Jutant et al. 2021). The leptin receptor has been reported to be distributed in many immune cells (Abella et al. 2017; Huertas et al. 2012) and can be divided into six subtypes based on cytoplasmic domains: obR-a, obR-b, obR-c, obR-d, obR-f, and obR-e (Houseknecht et al. 1998; Wang et al. 1996). The long isoform (obR-b) of the subtypes is the key receptor for transmitting leptin signaling (Chen et al. 1996). Although the long isoform obR (obR-b) has 300 amino acid sites without enzymatic activity in the cytoplasm (Lee et al. 1996; Huising et al. 2006), leptin binding to obR-b can signal by activating tyrosine phosphorylation of molecules in downstream pathways such as SHP2, STAT5, ERK, JAK2, and STAT3 (Björnholm et al. 2007). Leptin has been reported to increase airway inflammation in an allergic asthma mouse model without obesity (Zheng et al. 2018; Kurokawa et al. 2021). However, little is known about how leptin/obR signaling participates in the pathogenesis of obesity-related neutrophilic airway inflammation.

Macrophages are regarded as the predominant immune cells in lung diseases (Abdelaziz et al. 2020; Fehervari 2015). Alterations in macrophage function, especially macrophage phenotypes, were reported to contribute to asthma severity (Melgert et al. 2010; Draijer et al. 2017). Macrophages are classified as classically activated macrophages (M1) or alternatively activated macrophages (M2) (Fricker and Gibson 2017). Although M2-phenotype macrophages play an important role in allergic asthma, increasing evidence has indicated that M1-phenotype macrophages play an inflammatory role that affects the severity of nonallergic asthma (Kim et al. 2007). Obesity was reported to alter the function of immune cells (Mathis and Shoelson 2011). It was shown that M2-phenotype macrophages could be switched to pro-inflammatory M1-phenotype macrophages in adipose tissues (Chawla et al. 2011). We speculate that M1 macrophages may play an inflammatory role in the neutrophilic airways of obesity-related asthma. Therefore, it is very important to investigate the role of M1 macrophages in obesity-associated neutrophil airway inflammation.

To our knowledge, few studies have studied the inflammatory effects of leptin/obR signaling on the upregulation of M1 macrophage polarization in obesity-related neutrophilic airway inflammation. Immune-related airway inflammation is a characteristic feature of obesity-related asthma (Hammad and Lambrecht 2021). We explored the interaction between leptin/obR signaling and M1 macrophage polarization in obesity-related neutrophilic airway inflammation using a lipopolysaccharide (LPS)/ovalbumin (OVA) + OVA-treated obese mouse model, which has features similar to those of obesity-related asthma. The effects of leptin/obR signaling on obesity-related neutrophilic airway inflammation were reversed upon treatment with an obR antagonist (Allo-Aca) or absent in db/db mice (obR-b deficiency). Furthermore, our study indicated that JNK/STAT3/AKT signaling and CXCL2 production were significantly involved in the cellular activation of M1 macrophage induced by leptin/obR. We suggest that obesity-related elevation of leptin/obR signaling has pathogenic effects by increasing M1 macrophage polarization in obesity-related neutrophilic airway inflammation, and we provide a promising drug target for obesity-related asthma.

## Materials and methods

### Animal experiments

The animal experimental protocols followed the ARRIVE guidelines. The Medical Ethics Committee of Xiangya Hospital Central South University approved the study with approval number 201803692. Female C57BL/6 mice (6–8 weeks of age; 19–20 g, Laboratory Animal Center of Central South University (Changsha, China)) were kept under a 12:12-h light/dark cycle and fed water and food. A group of female mice received a high-fat diet (HFD) (60 Kca% as fat, Medicience Ltd., JS, CN) for 16 weeks before neutrophilic airway inflammation mouse model establishment according to previous protocols with minor modifications (Fang et al. 2018; Wilson et al. 2009; Kim et al. 2020). Briefly, female mice received 0.1 μg of LPS (InvivoGen, Estonia, France) and 100 μg of OVA (Grade V, Sigma, Missouri, USA) in 50 μl of PBS intratracheally under pentobarbital anesthesia on day 0 and day 7. On day 14, female mice were challenged with 5% OVA (Grade III, Sigma, Missouri, USA) aerosol for 40 min. On day 15, female mice were sacrificed for histological analysis and bronchoalveolar lavage fluid (BALF) collection. BALF was collected by lavage from the lungs with 0.6 ml of sterile PBS in a 1-ml syringe 10 times, and BALF cells were obtained after centrifuging the BALF at 400×*g* and 4 °C for 5 min.

Another group of female mice received a normal diet (lean mice) for 16 weeks, and then a neutrophilic airway inflammation mouse model was established. Another group of female mice was sensitized and challenged with an equal dosage of PBS as a control. Allo-Aca (GenScript, China) is a selective ObR antagonist peptide without agonistic activity (Otvos et al. 2011). In a separate set of experiments, female mice with diet-induced obesity were treated with Allo-Aca (0.1 mg/kg/day, dissolved in 100 μl of PBS) or PBS vehicle control (vehicle) by intraperitoneal injection, starting from the first day of LPS/OVA exposure until 2 h before OVA challenge on day 14. The potential role of obR was also examined in female obR-b-deficient (db/db) obese mice (Hunan SJA Laboratory Animal CO., LTD, Changsha, China).

### Mouse airway inflammation analysis

Neutrophilic airway inflammation was evaluated by lung tissue histology analysis using hematoxylin and eosin (H&E) (Lee et al. 2009), BALF inflammatory cell counts, and inflammatory cytokine levels in lung homogenates as previously described (Tan et al. 2019; Ke et al. 2019; Qu et al. 2017). Periodic acid-Schiff (PAS) staining was used to evaluate mucus hypersecretion in the airway (McMillan et al. 2002), which indicated airway obstruction.

### Experiments with primary bone marrow-derived macrophages (BMDMs)

Primary bone marrow-derived macrophages (BMDMs) were isolated and cultured as previously reported (Bai et al. 2021). Mature BMDMs were exposed to 20 ng/ml IFN-γ plus 50 ng/ml LPS in the presence or absence of leptin (2 µg/ml, Peprotech, New Jersey, USA) for 24 h before the assessment of M1 macrophage polarization. Mature BMDMs were exposed to 20 ng/ml IL-4 (Peprotech, New Jersey, USA) in the presence or absence of leptin (2 µg/ml) for 24 h before the assessment of M2 macrophage polarization. In some experiments, the leptin receptor antagonist Allo-Aca (1 mM) was included to verify the effects of the leptin receptor. In some experiments, synthetic inhibitors of STAT3 (Stattic, 100 nM, Selleck, Texas, USA), JNK (Sp600, 10 μM, Selleck, Texas, USA), or AKT (MK-2206, 10 μM, Selleck, Texas, USA) were used to verify the effects on M1 macrophages.

### Inflammatory cytokine detection

The levels of Th1/T helper 17 (Th17)-associated inflammatory cytokines (IFN-γ, TNF-α, IL-1β, IL-6, and IL-17A) were detected in lung homogenates and cell-free supernatants using a bead-based multiplex LEGENDplexTM Kit (BioLegend, San Diego, CA, US).

### Western blotting and lung immunofluorescence

Western blotting and lung immunofluorescence were performed as previously described (Zhang et al. 2021a). The first antibody (F4/80, obR, or iNOS) was used for probing in the lung section. The primary antibodies are listed in Additional file 1: Table S1.

### Enzyme-linked immunosorbent assay (ELISA)

The leptin concentration in serum was measured using a Mouse Leptin ELISA Kit (RayBiotech, Norcross, GA, USA) or a Human Leptin ELISA Kit (NeoBioscience Technology Co., Ltd, China). The CXCL2 concentration in the cell supernatant was measured using a Mouse CXCL2 ELISA Kit (RENJIEBIO Co, Ltd, China). The assays were carried out according to the manufacturer’s instructions.

### Reverse transcription‐polymerase chain reaction (RT-PCR)

RT‒PCR was performed using ChamQ Universal SYBR qPCR Master Mix (Vazyme, Nanjing, China). The primers for each target gene (Tsingke Biotechnology Co., Ltd., Beijing, China) are displayed in Additional file 3: Table S3.

### Cell viability assay

The CCK-8 assay was performed according to previously described methods (Zhao et al. 2016; Liu et al. 2016).

### Murine neutrophil isolation

Murine neutrophils were collected from murine bone marrow using centrifugation with discontinuous density solutions (Histopaque-1077 and Histopaque-1119) (Solarbio) according to previous studies (Zhang et al. 2022; Jiao et al. 2021).

### Neutrophil migration assay

Neutrophil migration was carried out in a Transwell system with a 5-μm polycarbonate membrane (Corning). The conditioned medium was centrifuged to remove cells and then placed at the bottom of the Transwell system. Neutrophils (2 × 10^5^ cells/100 μl) suspended in complete RPMI 1640 were added to the top of the Transwell system. The transwell system was incubated in 5% CO2 and at 37 °C for 2 h. Migrated neutrophils were counted using the chemotactic index according to previously described methods (Zhang et al. 2022; Czepielewski et al. 2012).

### Human samples

The diagnosis of asthma was established based on typical respiratory symptoms, a doctor’s diagnosis, and spirometry findings following the Global Initiative for Asthma (GINA) recommendations (Bousquet 2000). The exclusion criteria were defined as follows: (1) patients who had other immune system diseases; (2) patients who had other respiratory system diseases; (3) patients who had malignant tumors; and (4) patients who used antibiotics or systemic corticosteroids within 1 week. Adults including obese patients with asthma (OA, n = 14) and nonobese patients with asthma (NOA, n = 25) were recruited for our study. All participants provided informed written consent to participate in the study. The OA group (≥ 28 kg/m^2^) and the NOA group (< 24 kg/m^2^) were defined according to the BMI criteria for Chinese adults (Wang et al. 2021). Serum and induced sputum were obtained from patients with asthma. Informed consent was obtained from all patients. Cells were isolated from human-induced sputum as previously described (Kim et al. 2019). The study was approved by the Medical Ethics Committee of Xiangya Hospital with approval number 201803691, following the Code of Ethics of the World Medical Association.

### Flow cytometry

Cells from bone marrow, lung suspension, BALF, and human-induced sputum were prepared as previously described for flow cytometry (Zhang et al. 2021b). For cell surface staining, cells were stained with the antibody for 30 min at 4 °C. A list of the antibodies used in our study is displayed in Additional file 2: Table S2. Staining quantification was performed using a Cytek Dxp Athena flow cytometer. FlowJo software (version 10, Treestar, Ashland,USA) was used for analysis.

### Statistical analysis

Statistical analysis was performed using SPSS software (version 19, Chicago, IL, USA), and results were graphed using GraphPad Prism 9 (La Jolla, Calif). Continuous variables following a normal distribution are presented as the mean ± standard deviation (SD). Continuous variables with non-normal distribution are presented as median and quartile. If 2 groups were involved, a t test was used for analysis. If multiple groups were involved, one-way ANOVA was used for parametric analysis; otherwise, Bonferroni's post hoc test was used for nonparametric analysis. For correlation analysis, Pearson correlation tests were used for parametric data; otherwise, the Spearman r correlation test was performed for nonparametric data. P values were considered significant when P < 0.05.

## Results

### Increased airway inflammation in a female obesity-related neutrophilic airway inflammation mouse model

**Fig. 1:**
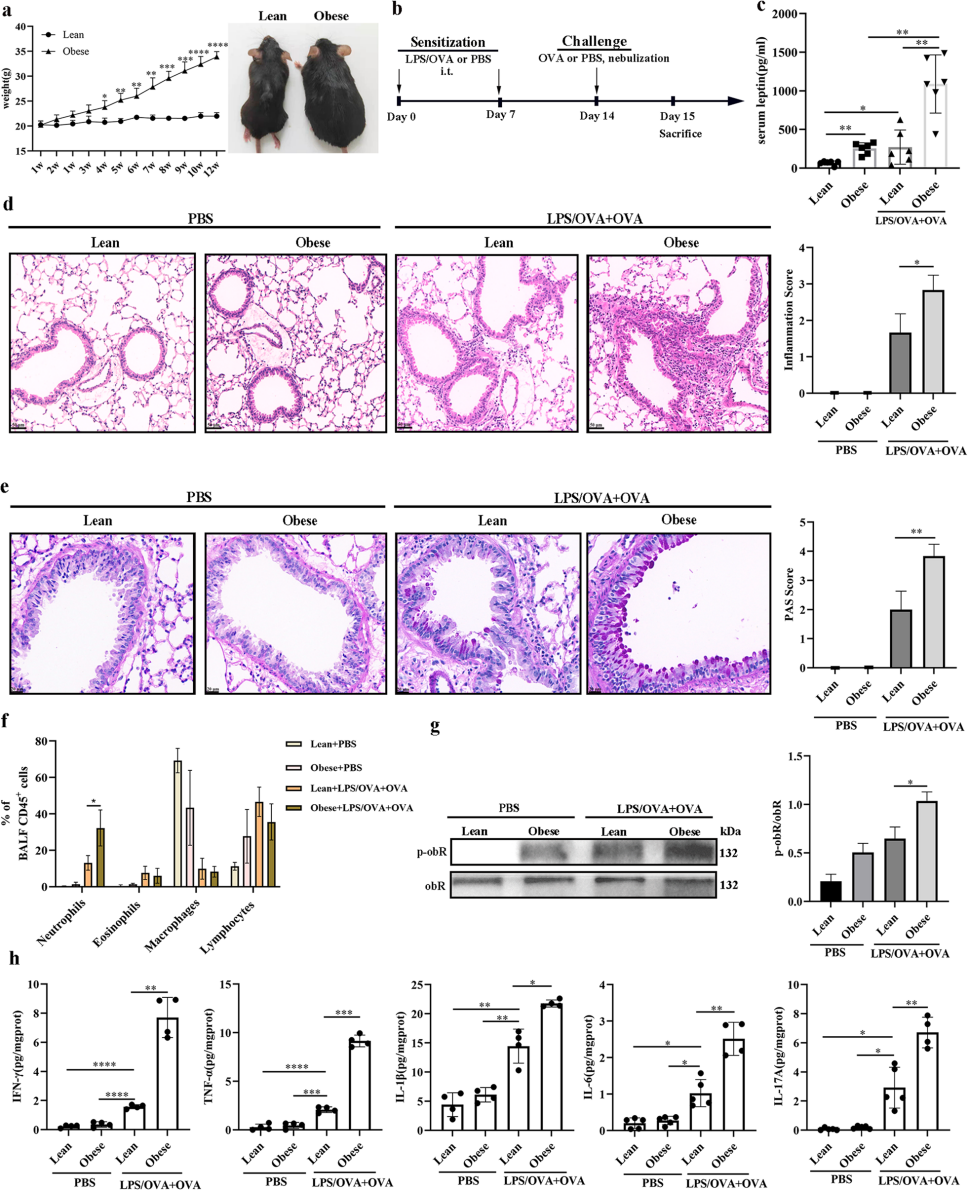
Increased airway inflammation in a female obesity-related neutrophilic airway inflammation mouse model. **a** Comparison of body weight changes between lean and obese female mice. **b** Flow chart for the generation of the neutrophilic airway inflammation mouse model. **c** Comparison of serum leptin levels between female mice. **d** Representative H&E-stained histological lung sections and a bar graph showing the inflammation scores. Scale bar = 50 μm. **e** Representative PAS-stained histological lung sections and a bar graph showing the PAS scores. Scale bar = 20 μm. **f** Neutrophil, eosinophil, macrophage, and lymphocyte percentages in BALF were measured by flow cytometry. **g** Western blot analysis of obR-b phosphorylation in lung tissues. The displayed protein expression value is the ratio of phosphorylated protein to total protein. **h** Levels of inflammatory cytokines (IFN-γ, TNF-α, IL-1β, IL-6, and IL-17A) in lung homogenate. The data are expressed as the means ± SDs. *P < 0.05, **P < 0.01, ***P < 0.001, ****P < 0.0001. i.t., intratracheal; i.p., intraperitoneal. For a-e, n = 6 female mice per group; for g, n = 3 female mice per group; for h, n = 4–6 female mice per group

Female mice fed a high-fed diet (HFD) gained body weight over the observed period and were classified as the obese group. Female mice fed a normal control diet slightly gained body weight over the observed period and were classified as the lean group. The body weight of the obese group was remarkably increased compared with that of the lean group (Fig. 1a). We used LPS/OVA sensitization and OVA challenge (LPS/OVA + OVA) to establish a neutrophilic airway inflammation mouse model in female obese or lean mice, as described in Fig. 1b. Female obese mice (253.6 ± 73.67 pg/ml) had higher serum leptin levels than female lean mice (67.19 ± 27.56 pg/ml) (Fig. 1c), indicating that obesity is related to hyperleptinemia. Additionally, serum leptin levels in female mice after LPS/OVA + OVA treatment were significantly higher than those in female mice before LPS/OVA + OVA treatment, especially in female obese mice (Fig. 1c). The plasma insulin level was significantly higher in obese mice than that in lean mice, indicating insulin resistance in obese mice (Additional file 5). Besides, obese or lean mice slightly reduced weight after LPS/OVA+OVA treatment, but the difference was not significant (Additional file 6a–b). H&E staining showed peribronchial infiltration of inflammatory cells. PAS staining showed mucus secreted by goblet cells, which was related to airway obstruction. LPS/OVA sensitization plus OVA challenge induced obvious peribronchial inflammation (H&E) and goblet cell hyperplasia (PAS). In the LPS/OVA + OVA-treated mouse model, female obese mice had higher levels of peribronchial inflammation (H&E) and goblet cell hyperplasia (PAS) than female lean mice (Fig. 1d-e). Furthermore, flow cytometry analysis revealed high neutrophil percentages and low percentages of eosinophils in the BALF of LPS/OVA + OVA-treated female mice, suggesting the successful establishment of neutrophilic airway inflammation, as previously reported (Tan et al. 2019). In the LPS/OVA + OVA-treated mouse model, female obese mice had higher levels of neutrophils in BALF than female lean mice (Fig. 1f). Moreover, the phosphorylation levels of the long isoform obR (obR-b) were higher in LPS/OVA + OVA-treated female obese mice than in LPS/OVA + OVA-treated female lean mice (Fig. 1g), indicating activation of obR-b in the female obese mouse model. Furthermore, the levels of Th1/17 inflammatory cytokines (IFN-γ, TNF-α, IL-1β, IL-6, and IL-17A) were elevated in lung tissues in response to LPS/OVA + OVA stimulation and markedly increased in the LPS/OVA + OVA-treated female obese mice (Fig. 1h). Thus, our results indicated that neutrophilic airway inflammation was significantly increased in the LPS/OVA + OVA-treated female obese murine model, and the leptin/obR axis may be associated with the pathogenesis of obesity-related neutrophilic airway inflammation in females.

### Increased M1-phenotype macrophages in a female obesity-related neutrophilic airway inflammation mouse model

**Fig. 2:**
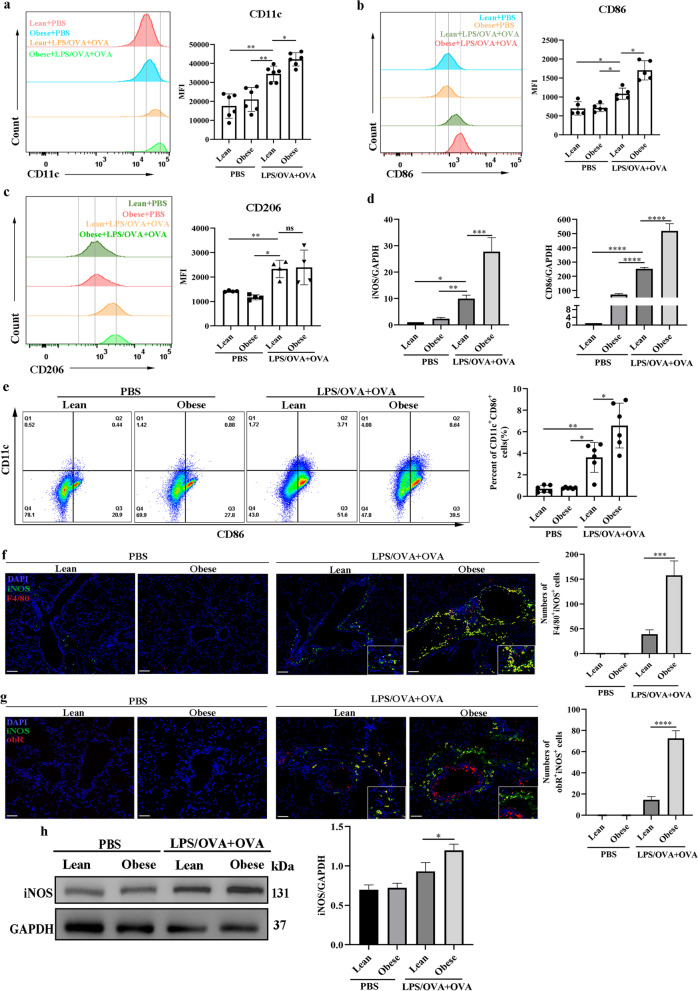
Increased M1-phenotype macrophages in a female obesity-related murine neutrophilic airway inflammation mouse model. **a** Representative histogram of CD11c (M1) expression (left) and a bar graph showing the quantitative analysis of CD11c (right) in BALF. **b** Representative histogram of CD86 (M1) expression (left) and a bar graph showing the quantitative analysis of CD86 (right) in BALF. **c** Representative histogram of CD206 (M2) expression (left) and a bar graph showing the quantitative analysis of CD206 (right) in BALF. **d** Real-time PCR assessment of M1-associated gene (iNOS and CD86) mRNA levels normalized to those of GAPDH. **e** Representative flow cytometry analysis of M1 macrophages (CD11c^+^CD86^+^) (gating in macrophages) (left) and quantitative analysis of the M1 macrophage number (right) in lung single-cell suspensions. **f** Representative immunofluorescence staining of iNOS (green) and F4/80^+^ (red) in lung sections (×100). DAPI (blue) was used for nuclear visualization. Scale bar = 100 μm. **g** Representative immunofluorescence staining of iNOS (green) and obR (red) in lung sections (×200). DAPI (blue) was used for nuclear visualization. Scale bar = 50 μm. **h** Western blot analysis of iNOS in lung tissues. The displayed protein expression value is the ratio of iNOS protein to total protein. Data are expressed as the means ± SDs. *P < 0.05, **P < 0.01, ***P < 0.001, ****P < 0.0001. For **a** and **e**, n = 6 female mice per group; for **b**, n = 5 female mice per group; for **c**, **d**, n = 4–6 female mice per group; for **f**–**h**, n = 3 female mice per group

We detected M1 phenotype surface markers (CD11c, CD86) and an M2 phenotype surface marker (CD206) (Lu et al. 2018; Gambaro et al. 2018) in BALF to explore macrophage polarization in the female mouse model. LPS/OVA + OVA treatment significantly increased the production of CD11c (Fig. 2a), CD86 (Fig. 2b), and CD206 (Fig. 2c) in BALF. Furthermore, there was a significant increase in the production of CD11c (Fig. 2a) and CD86 (Fig. 2b) in LPS/OVA + OVA-treated female obese mice as compared with LPS/OVA + OVA-treated female lean mice, while LPS/OVA + OVA-treated female obese and lean mice did not differ significantly in the production of CD206 (Fig. 2c), indicating that M1 macrophage polarization was elevated in BALF from female mice with obesity-related neutrophilic airway inflammation. In addition, the qRT‒PCR results showed that the LPS/OVA + OVA-treated obese group showed the highest levels of M1 macrophage-associated genes (iNOS and CD86) (Fig. 2d). Furthermore, there was a significant increase in the number of M1 (CD11c^+^CD86^+^) macrophages in lung single-cell suspensions from female obese mice compared with those form female lean mice after LPS/OVA + OVA treatment (Fig. 2e). Coimmunostaining of lung sections demonstrated that iNOS was predominantly expressed in F4/80^+^ cells (infiltrated macrophages) in LPS/OVA + OVA-treated female obese mice compared to LPS/OVA + OVA-treated female lean mice (Fig. 2f). In addition, coimmunostaining of lung sections showed that the number of obR^+^iNOS^+^ cells in the lungs was higher in LPS/OVA + OVA-treated female obese mice than in LPS/OVA + OVA-treated female lean mice (Fig. 2g), suggesting that obR^+^ cells in the LPS/OVA + OVA-treated female obese murine model exhibited a more M1-like phenotype. In addition, iNOS exhibited the most protein activation in the LPS/OVA + OVA-treated female obese mice (Fig. 2h). Collectively, our results suggested that M1 macrophage polarization was enhanced in the female mice with obesity-related neutrophilic airway inflammation, and leptin/obR signaling may affect M1 macrophages.

### Allo-Aca inhibited M1 macrophage polarization and obesity-related murine neutrophilic airway inflammation in a female obese mouse model

**Fig. 3:**
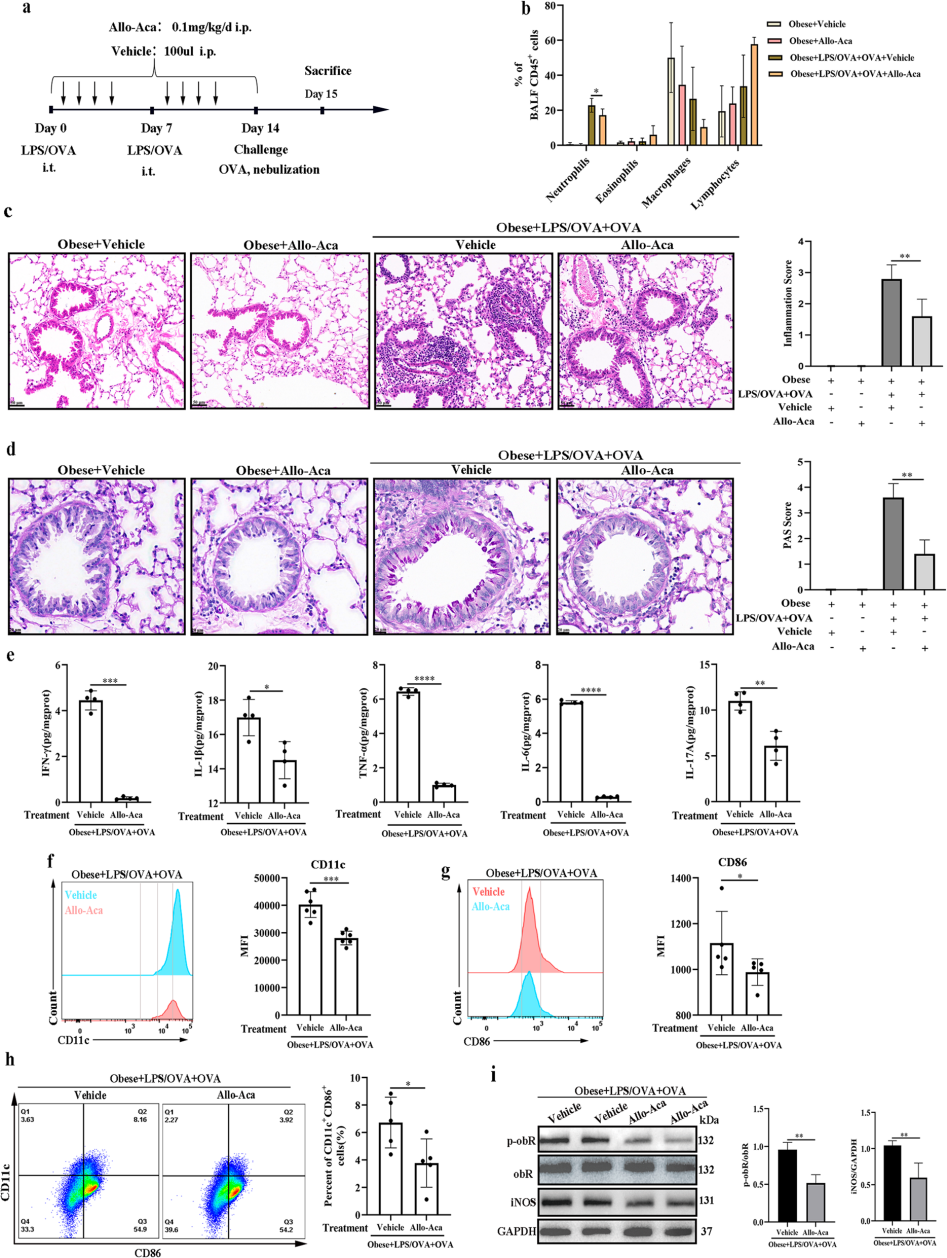
Allo-Aca inhibited M1 macrophage polarization and obesity-related murine neutrophilic airway inflammation in a female obese mouse model. **a** Flow chart for Allo-Aca or vehicle administration in a female obesity-related neutrophilic airway inflammation mouse model. **b** Neutrophil, eosinophil, macrophage, and lymphocyte percentages in BALF. **c** Representative H&E-stained histological lung sections and a bar graph showing the inflammation scores. Scale bar = 50 μm. **d** Representative PAS-stained histological lung sections and a bar graph showing the PAS scores. Scale bar = 20 μm. **e** The levels of inflammatory cytokines (IFN-γ, TNF-α, IL-1β, IL-6, and IL-17A) in lung homogenates. **f** Representative histogram of CD11c (M1) expression (left) and quantitative analysis of CD11c (right) expression in BALF. **g** Representative histogram of CD86 (M1) expression (left) and quantitative analysis of CD86 (right) expression in BALF. **h** Representative flow cytometry analysis to detect M1 macrophages (CD11c^+^CD86^+^) (gating in macrophages) (left) and quantification of M1 macrophage number (right) in lung single-cell suspensions. **i** Western blot analysis of obR-b phosphorylation and iNOS expression in lung tissues. The relative expression of protein displayed the ratio of phosphorylated protein or iNOS protein to total protein. The data are expressed as the means ± SDs. *P < 0.05, **P < 0.01, ***P < 0.001, ****P < 0.0001. i.t., intratracheal; i.p., intraperitoneal. For **a**–**d**, n = 4–6 female mice per group; for **e**, n = 4 female mice per group; for **f**, n = 6 female mice per group; for **g**, **h**, n = 5 female mice per group; for **i**, n = 3 female mice per group

We next explored the role of obR in a female obesity-related neutrophilic airway inflammation mouse model using an obR antagonist (Allo-Aca), as illustrated in Fig. 3a. The plasma insulin level was slightly higher in LPS/OVA+OVA-treated obese mice compared to LPS/OVA+OVA+Allo-Aca-treated obese mice, but the differences were not significant (Additional file 5). Allo-Aca treatment reduced the numbers of neutrophils in BALF (Fig. 3b), peribronchial inflammation (H&E) (Fig. 3c), goblet cell hyperplasia (PAS) (Fig. 3d), and the levels of Th1/17-related inflammatory cytokines (Fig. 3e) in LPS/OVA + OVA-treated female obese mice, indicating that Allo-Aca inhibited the obese-related neutrophilic airway inflammation. The levels of CD11c (Fig. 3f) and CD86 (Fig. 3g) in BALF and the number of M1-phenotype macrophages (CD11c^+^CD86^+^) (Fig. 3h) in lung single-cell suspensions were also reduced in LPS/OVA + OVA-treated female obese mice after Allo-Aca treatment, suggesting that Allo-Aca inhibited M1 macrophage polarization in the female obese murine model. As expected, administration of Allo-Aca to LPS/OVA + OVA-treated female obese mice resulted in reduced phosphorylation levels of obR-b and protein levels of iNOS (Fig. 3i). Taken together, these results suggested that the obR inhibitor reduced M1 macrophage polarization and further alleviated neutrophilic airway inflammation in a female obesity-related neutrophilic airway inflammation murine model.

### M1 macrophage polarization and obesity-related neutrophilic airway inflammation were reduced in a female db/db murine model

**Fig. 4:**
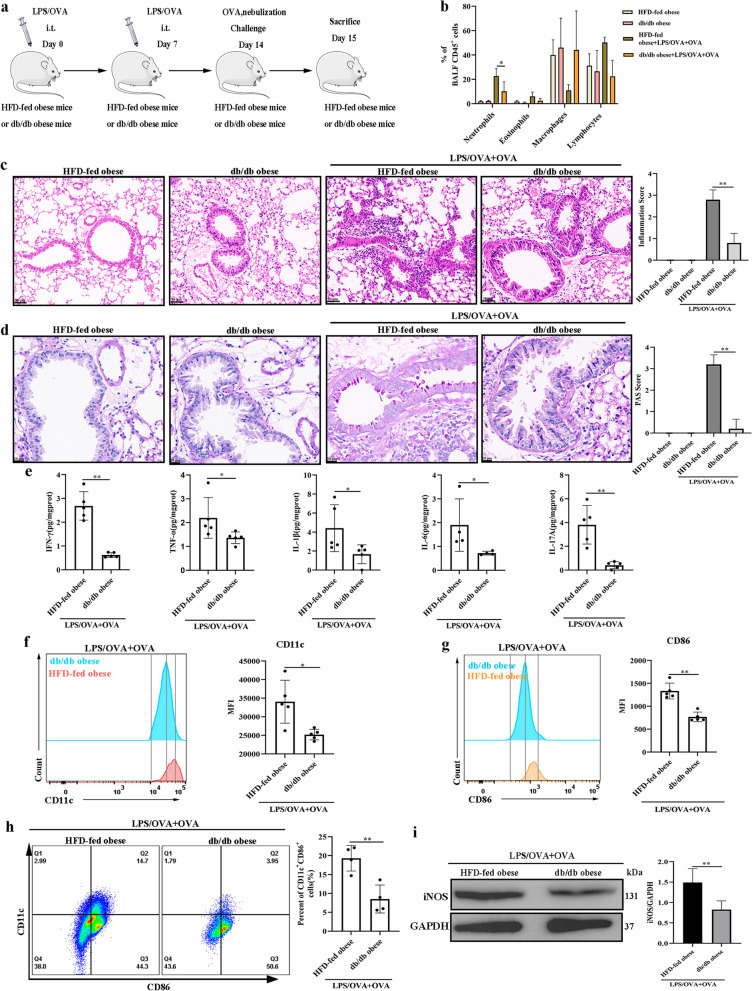
M1 macrophage polarization and obesity-related neutrophilic airway inflammation were reduced in a female db/db murine model. **a** Flow chart for the generation of a female obesity-related neutrophilic airway inflammation mouse model. **b** Neutrophil, eosinophil, macrophage, and lymphocyte percentages in BALF. **c** Representative H&E-stained histological lung sections and a bar graph showing the inflammation scores. Scale bar = 50 μm. **d** Representative PAS-stained histological lung sections and a bar graph showing the PAS scores. Scale bar = 20 μm. **e** The levels of inflammatory cytokines (IFN-γ, TNF-α, IL-1β, IL-6, and IL-17A) in lung homogenates. **f** Representative histogram of CD11c (M1) expression (left) and quantitative analysis of CD11c (right) expression in BALF. **g** Representative histogram of CD86 (M1) expression (left) and the quantitative analysis of CD86 (right) expression in BALF. **h** Representative flow cytometry analysis of M1 macrophages (CD11c^+^CD86^+^) (gating on macrophages) (left) and quantitative analysis of the M1 macrophage number (right) in lung single-cell suspensions. **i** Western blot analysis of iNOS in lung tissues; GAPDH was used as a loading control. The displayed protein expression value is the ratio of iNOS protein to total protein. The data are expressed as the means ± SDs. *P < 0.05, **P < 0.01, ***P < 0.001, ****P < 0.0001. i.t., intratracheal; i.p., intraperitoneal. For **b**–**d** and **f**, **g**, n = 5 female mice per group; for **e**, n = 4–5 female mice per group; for **h**, n = 4 female mice per group; for i, n = 3 female mice per group

To explore whether obR-b mediates the function of leptin, female db/db mice, which were considerably obese and lacked the long obR isoform (obR-b) (Lu et al. 2006), were used to generate a neutrophilic airway inflammation mouse model, as shown in Fig. 4a. After LPS/OVA + OVA treatment, female db/db obese mice showed fewer neutrophils in BALF (Fig. 4b), less peribronchial inflammation (H&E) (Fig. 4c), less goblet cell hyperplasia (PAS) (Fig. 4d), and decreased levels of Th1/17 cytokines (Fig. 4e) than female HFD-fed obese mice, indicating that there was less neutrophilic airway inflammation in the female db/db mice. In addition, the production of CD11c (Fig. 4f) and CD86 (Fig. 4g) in BALF and the number of M1 (CD11c^+^CD86^+^) macrophages in lung single-cell suspensions (Fig. 4h) were also decreased in female db/db obese mice compared to female HFD-fed obese mice after LPS/OVA + OVA treatment, indicating that female db/db mice had less M1 macrophage polarization. The changes in M1 macrophage polarization were further supported by Western blot analyses (Fig. 4i). Collectively, our study showed that obR-b is the critical receptor that participates in leptin/obR-mediated M1 macrophage polarization and obesity-related neutrophilic airway inflammation in females.

### The mechanisms by which leptin affects M1 macrophage polarization

**Fig. 5:**
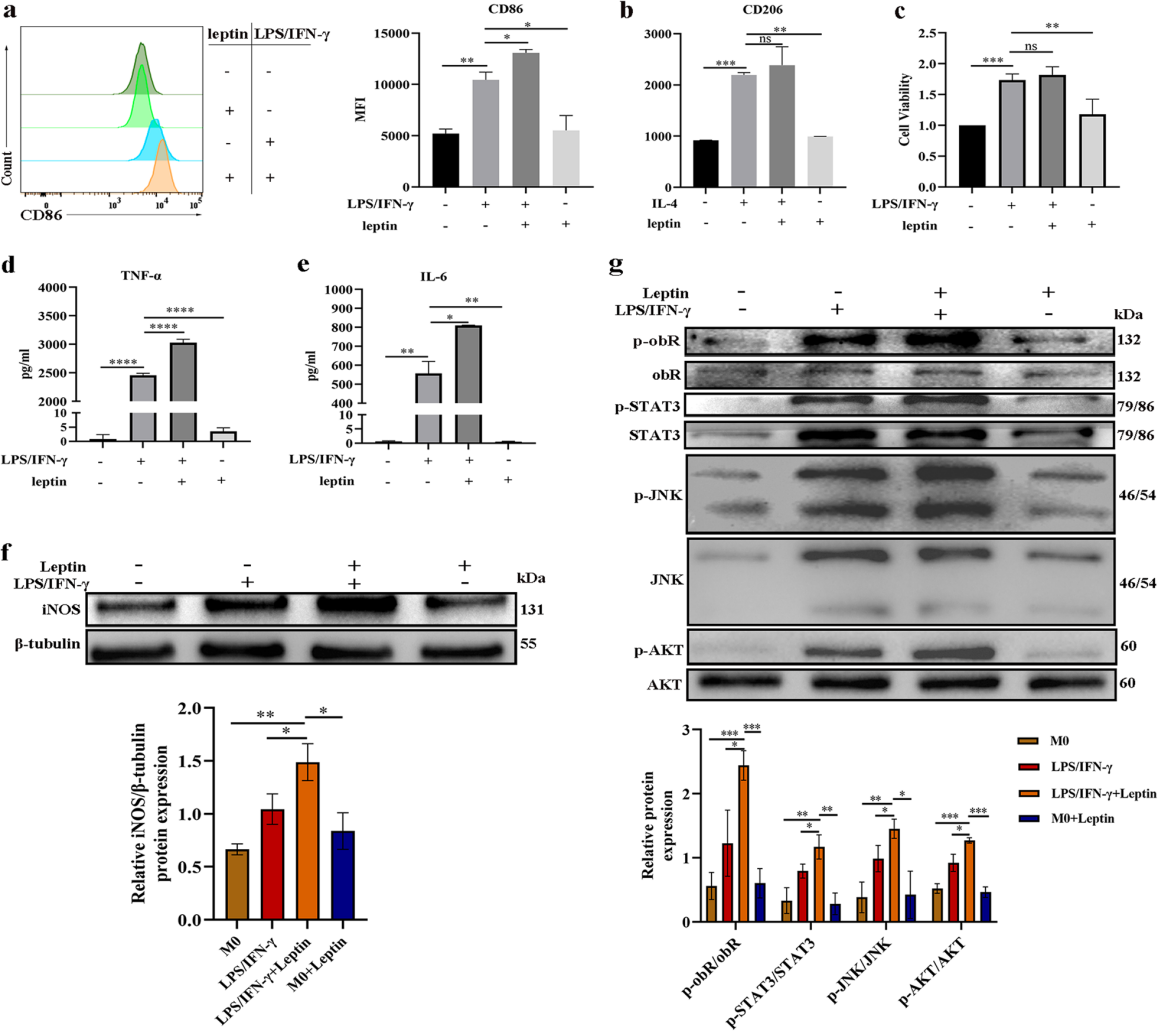
The mechanisms by which leptin affects M1 macrophage polarization. **a** Representative histogram of CD86 expression (left) and quantitative analysis of CD86 (right) expression in BMDMs. **b** Quantitative analysis of CD206 expression in BMDMs. **c** Cell viability. **d, e** Levels of inflammatory cytokines (TNF-α and IL-6) in cell-free supernatants of BMDMs. **f** Western blot analysis of iNOS in BMDMs and β-tubulin was used as a loading control. The displayed protein expression value is the ratio of iNOS protein to total protein. **g** Western blot analysis of the phosphorylation levels of obR-b and members of the downstream JNK/STAT3/AKT pathways in BMDMs. The displayed protein expression value is the ratio of phosphorylated protein to total protein. *P < 0.05, **P < 0.01, ***P < 0.001, ****P < 0.0001, each experiment was performed three times

BMDMs were used to explore the mechanism by which the leptin/obR axis regulates M1 macrophage polarization. LPS/IFN-γ serve as potent stimulators of M1 macrophage polarization (Murray 2017). The production of CD86 in LPS/IFN-γ-induced BMDMs (M1 macrophages) was greater than that in unstimulated BMDMs (Fig. 5a), suggesting the successful induction of M1 macrophages. Leptin alone did not show a significant ability to induce M1 macrophage polarization. Moreover, compared to LPS/IFN-γ-induced BMDMs, BMDMs costimulated with leptin and LPS/IFN-γ showed higher production of CD86 (Fig. 5a), suggesting that leptin synergized with LPS/IFN-γ to increase the levels of M1 macrophage polarization. However, the production of CD206 showed no significant difference between IL-4-induced BMDMs (M2 macrophages) with and without leptin stimulation (Fig. 5b), indicating that the effect of leptin on M2 macrophage polarization was not obvious. In addition, the cell viability of LPS/IFN-γ-induced BMDMs was greater than that of BMDMs without stimulation or with leptin stimulation alone (Fig. 5c), but there was no obvious difference between LPS/IFN-γ-induced BMDMs with and without leptin stimulation (Fig. 5c). The concentrations of inflammatory cytokines (TNF-α and IL-6) in cell-free supernatants were higher for BMDMs costimulated with LPS/IFN-γ and leptin than for BMDMs stimulated with LPS/IFN-γ (Fig. 5d, e), reflecting the inflammatory role of leptin on M1 macrophages. Similarly, Western blot analysis revealed that leptin synergized with LPS/IFN-γ to significantly increase the levels of iNOS (Fig. 5f) in BMDMs. In addition, leptin synergized with LPS/IFN-γ to significantly increase the phosphorylation levels of obR-b (Fig. 5g) in BMDMs, showing that obR-b was activated in M1 macrophages after leptin stimulation. Regarding downstream pathways, leptin synergized with LPS/IFN-γ to significantly increase the phosphorylation of STAT3, JNK, and AKT in BMDMs (Fig. 5g). Altogether, our results indicated that leptin synergized with LPS/IFN-γ to increase M1 macrophage polarization and the phosphorylation levels of obR-b and members of the JNK/STAT3/AKT pathways in BMDMs.

### obR and pathway inhibitors reversed the effects of leptin on LPS/IFN-γ-induced M1 macrophages

**Fig. 6:**
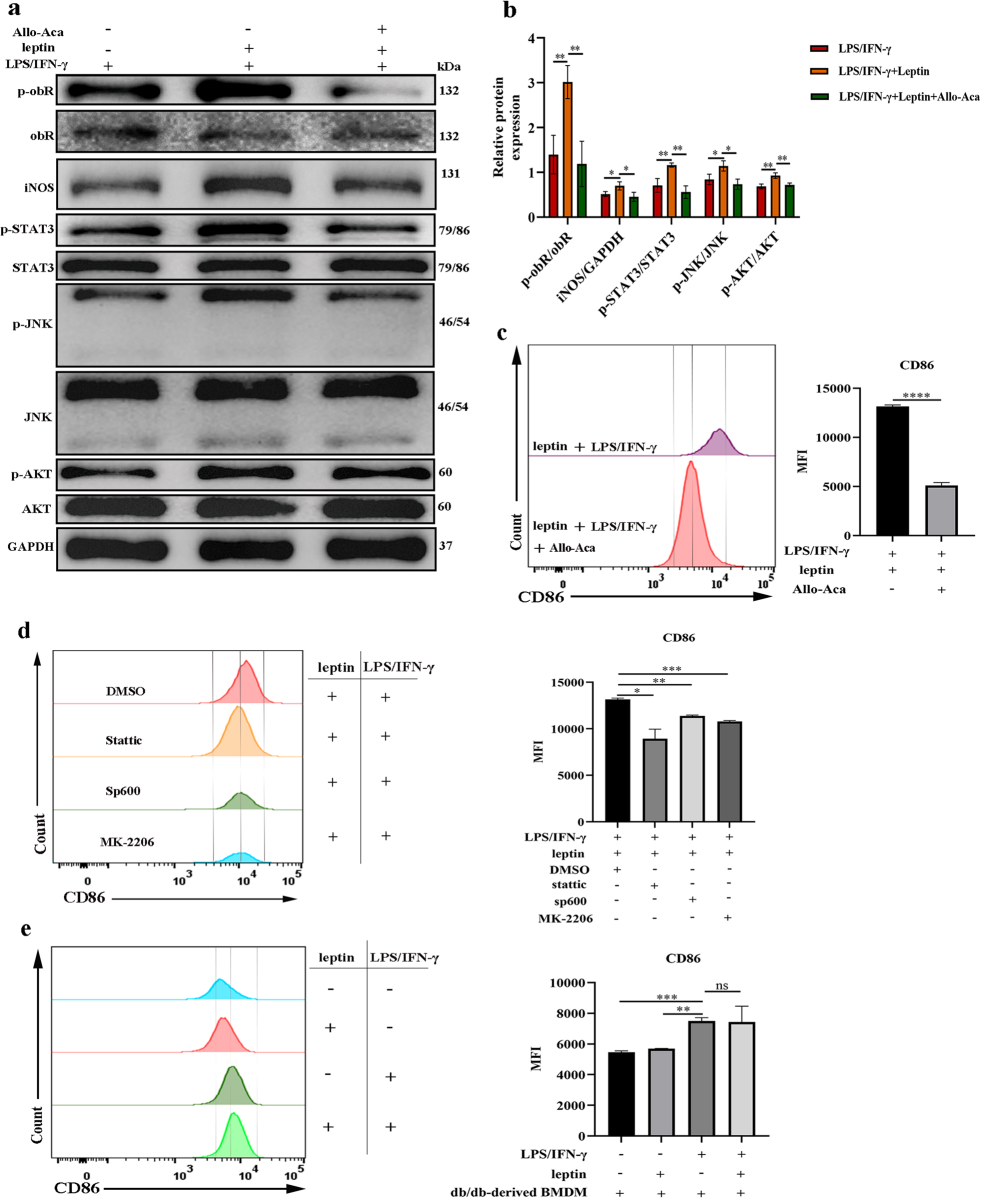
Signal inhibitors reversed the effects of leptin on LPS/IFN-γ-induced M1 macrophages. **a** Western blot analysis of iNOS and phosphorylation levels of obR-b and members of the downstream JNK/STAT3/AKT pathways in BMDMs. **b** The displayed protein expression value is the ratio of phosphorylated quantitative protein or iNOS protein to total protein. **c** Quantitative analysis of CD86 in BMDMs treated with or without Allo-Aca. **d** Quantitative analysis of CD86 in BMDMs treated with or without different synthetic inhibitors. **e** Representative histogram of CD86 expression (left) and quantitative analysis of CD86 (right) expression in female db/db mouse-derived BMDMs. *P < 0.05, **P < 0.01, ***P < 0.001, ****P < 0.0001, each experiment was performed three times

We further examined whether the effects of leptin on LPS/IFN-γ-induced M1 macrophage polarization occurred in an obR -mediated manner. Western blot analysis revealed that the obR inhibitor Allo-Aca markedly decreased the levels of phosphorylated obR-b and iNOS in BMDMs costimulated with leptin and LPS/IFN-γ (Fig. 6a, b). The phosphorylation levels of STAT3, JNK, and AKT in BMDMs costimulated with leptin and LPS/IFN-γ were also decreased after Allo-Aca treatment (Fig. 6a). Allo-Aca also decreased the production of CD86 in BMDMs costimulated with leptin and LPS/IFN-γ (Fig. 6c). Considering the changes in cell signaling, synthetic inhibitors of STAT3 (Stattic), JNK (Sp600), or AKT (MK-2206) were administered, resulting in decreased production of CD86 in BMDMs costimulated with leptin and LPS/IFN-γ (Fig. 6d). Next, flow cytometry revealed that there was no obvious difference in the production of CD86 between LPS/IFN-γ-stimulated db/db mouse-derived BMDMs with and without leptin stimulation (Fig. 6e). These results indicated that the effects of leptin on M1 macrophages relied on the activation of the obR and JNK/STAT3/AKT pathways.

### The effects of leptin/obR signaling on neutrophil chemotaxis

**Fig. 7:**
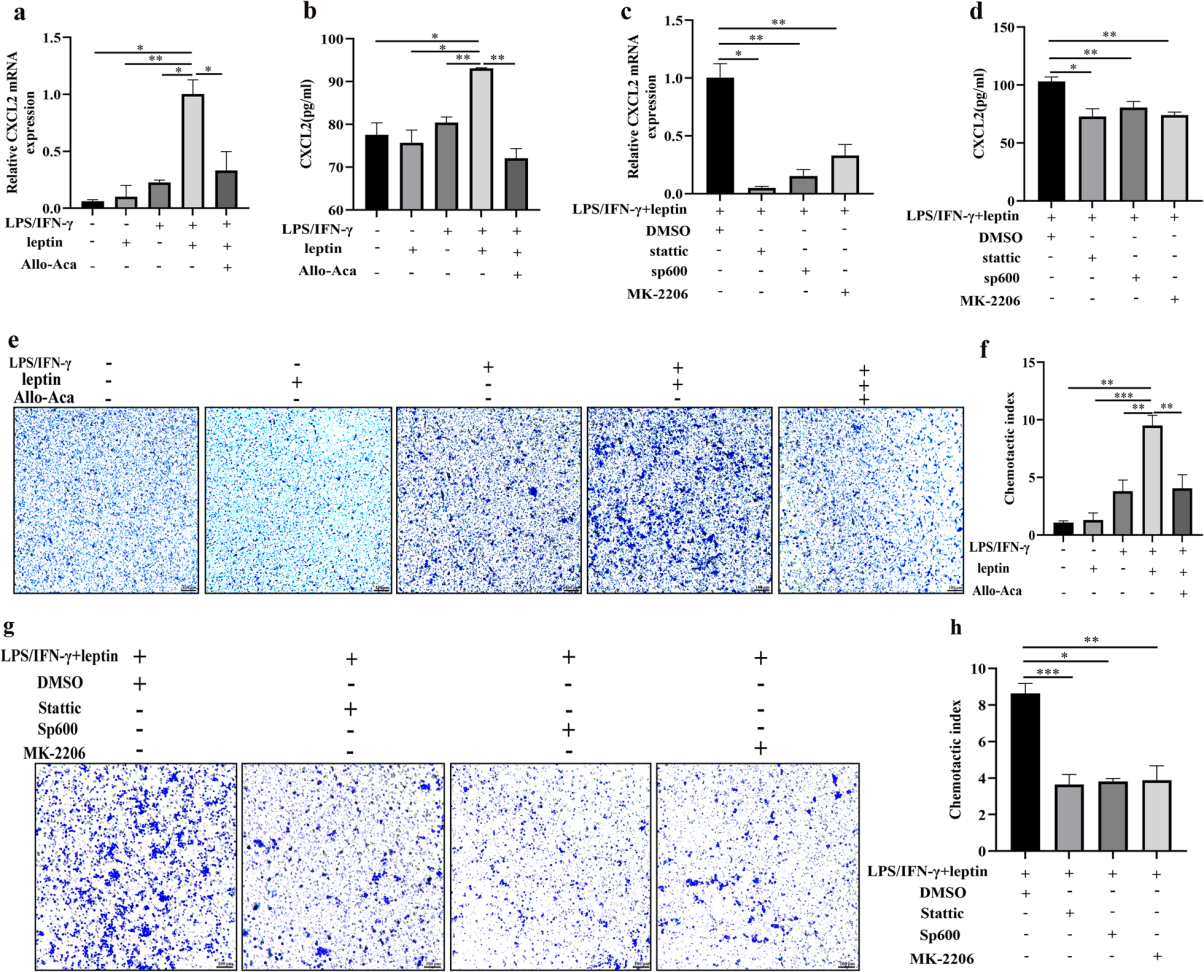
The effects of leptin/obR signaling on neutrophil chemotaxis. **a** Real-time PCR of CXCL2 mRNA levels normalized to those of GAPDH. **b** CXCL2 protein levels were analyzed by ELISA. **c** Real-time PCR of CXCL2 mRNA levels normalized to those of GAPDH. **d** CXCL2 protein levels were analyzed by ELISA. **e** Transwell assay to detect neutrophil chemotaxis (×100). **f** Chemotactic index of neutrophils. **g** Transwell assay to detect neutrophil chemotaxis (×100). **h** Chemotactic index of neutrophils. Each experiment was performed three times. *P < 0.05, **P < 0.01, ***P < 0.001, ****P < 0.0001

Previous studies indicated that neutrophils are chemoattracted and penetrated by macrophages in a CXCL2-dependent manner (Tsutsui et al. 2018; Filippo et al. 2013). In our present study, the qRT‒PCR showed that leptin significantly upregulated the mRNA levels of CXCL2 in LPS/IFN-γ-induced M1 macrophages (Fig. 7a). Similarly, ELISA showed that leptin significantly increased the protein expression of CXCL2 in the culture supernatants of LPS/IFN-γ-induced M1 macrophages (Fig. 7b). CXCL2 production in leptin-treated M1 macrophages was significantly abrogated after treatment with the obR antagonist Allo-Aca (Fig. 7a, b). In addition, the inhibition of STAT3, JNK or AKT significantly decreased the mRNA levels of CXCL2 and the expression of CXCL2 in the culture supernatants of M1 macrophages induced by leptin and LPS/IFN-γ (Fig. 7c, d). To explore the effect of leptin on the stimulation of LPS/IFN-γ-induced M1 macrophage recruitment of bone marrow-derived neutrophils, neutrophils were cocultured with conditioned medium for transwell analysis for 2 h. The cell culture supernatant in the leptin-treated M1 macrophage group significantly promoted neutrophil migration, whereas the effect was reversed in the Allo-Aca group (Fig. 7e, f), suggesting that extracellular effects of the leptin/obR axis on M1 macrophages promoted neutrophil recruitment. In addition, the extracellular effects of leptin on M1 macrophage-induced neutrophil recruitment were also reduced by inhibition of STAT3, JNK or AKT (Fig. 7g, h). These results showed that the leptin/obR axis increased the production of CXCL2 in M1 macrophages and played a crucial role in neutrophil recruitment through the JNK/STAT3/AKT pathways.

### Leptin concentration and M1 macrophage polarization in obese and nonobese patients with asthma

**Fig. 8:**
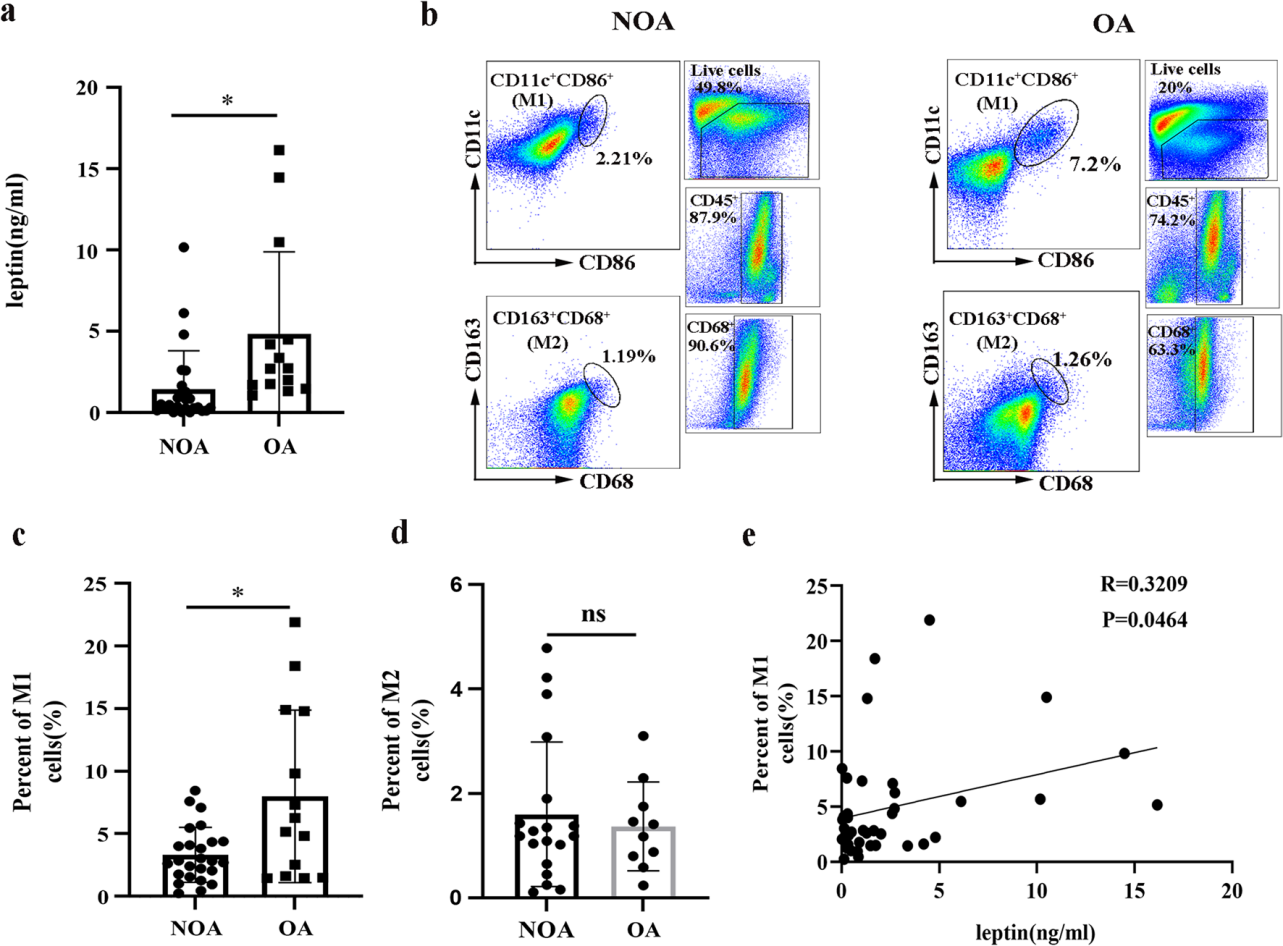
Leptin concentration and M1 macrophage polarization in obese and nonobese patients with asthma. **a** The level of leptin in the serum of OA patients (n = 14) and NOA patients (n = 25). **b** Representative macrophage gating strategy for the induced sputum of OA and NOA subjects. **c** Bar graph showing the number of M1 macrophages (CD11c^+^CD86^+^) in the induced sputum of OA and NOA subjects. **d** Bar graph showing the number of M2 macrophages (CD68^+^CD163^+^) in the induced sputum of OA and NOA subjects. **e** Correlation between serum leptin and M1 macrophage levels in induced sputum. The data are expressed as the means ± SDs. *P < 0.05, **P < 0.01, ***P < 0.001, ****P < 0.0001

The clinical features of the study subjects are displayed in Table 1. The body mass index (BMI) (P < 0.0001) and neutrophil counts in induced sputum (P = 0.0048) of the OA group were significantly higher than those of the NOA group (Table 1), suggesting that more neutrophils had infiltrated in the group with obesity-related airway inflammation. We detected the level of leptin in the serum of patients with asthma by ELISA. As expected, the serum leptin concentration was significantly higher in the OA group than in the NOA group (Fig. 8a). Furthermore, we detected macrophage phenotypes in the induced sputum of asthma patients by flow cytometry. The gating strategies are displayed in Fig. 8b. There was a significant increase in the number of M1 macrophages (CD11c^+^CD86^+^) in the induced sputum of the OA group compared to the NOA group (Fig. 8c). However, the two groups did not differ significantly in the number of M2 macrophages (CD68^+^CD163^+^) in induced sputum (Fig. 8d). Moreover, the number of M1 macrophages in induced sputum was positively correlated with the level of serum leptin (R = 0.3209, P = 0.0464, Fig. 8e). All the results suggested that the correlation between leptin and M1 macrophages may be related to neutrophil airway inflammation in obesity-associated asthma.

**Table 1:** Clinical characteristics of the subjects

	NOA	OA	P value
No. of patients (n)	25	14	
Age (y)	49 ± 10.5	54.43 ± 10.77	0.133
Female sex (%)	64	64.3	0.224
BMI	21.43 ± 1.71	30.35 ± 1.83	< 0.0001
Atopy (%)	36	28.58	0.289
FEV_1_ (%)	80.84 ± 20.7	73.76 ± 18.7	0.299
FEV_1_/FVC	81.72 ± 15.92	79.77 ± 15.58	0.715
Smoking history (never/smoker), no. (%)	76/24	71.4/28.6	0.522
ACT	16.92 ± 3.89	18.14 ± 4.66	0.386
Sputum Neu (%)	29.27 ± 20.91	56.55 ± 28.58	0.0048
Sputum Eso (%)	16.20 [5.97, 31.35]	16.4 ± 14.29	0.418
Peripheral blood			
WBC (× 10^9/L)	6.16 ± 1.51	6.48 ± 2.34	0.603
Neutrophils (%)	48.87 ± 16.25	50.49 ± 14.31	0.759
Eosinophils (%)	4.5 ± 4.78	3.79 ± 2.57	0.607

Categorical variables are represented as n (%), and continuous variables following a normal distribution are represented as the mean ± SD, and continuous variables with non-normal distribution are represented as median and quartile. NOA: nonobese asthma; OA: obese asthma; BMI: body mass index; FEV_1_: forced exhaled volume at 1 s; FVC: forced vital capacity; ACT: asthma control test; Neu: neutrophils; Eos: eosinophils

## Discussion

Asthma is a chronic inflammatory disorder of the airways driven by several immune cells (Zasłona et al. 2014). Glucocorticoids are the cornerstone of asthma treatment, while obesity-related asthma exhibits poor responsiveness to glucocorticoids, and thus, treatment is still very challenging (Peters et al. 2018). Airway inflammation in allergic asthma is predominantly driven by a Th2-dependent response, which is required for eosinophil infiltration (Bosnjak et al. 2011; Caminati et al. 2018). Obesity-related asthma, also known as “noneosinophilic asthma”, has been suggested to be related to non-Th2 responses (Moore et al. 2014; Scott et al. 2011; Leiria et al. 2015). Non-Th2 responses, including Th1/Th17 responses (related to the cytokines IFN-γ, TNF-α, IL-1β, IL-6, and IL-17A), have been implicated in neutrophilic airway inflammation in neutrophilic asthma (Sze et al. 2020). Compared with nonobese asthmatic patients, obesity-associated asthmatic patients had a higher level of neutrophils in sputum (Telenga et al. 2012). Leptin is a 167-amino-acid protein that is mainly produced by adipocytes in adipose tissue (Luis et al. 2009). Obesity features high serum levels of leptin and is related to chronic low-grade inflammation (Bantulà et al. 2021; Choi et al. 2020). Studies have indicated that high levels of leptin are associated with many immune and inflammatory diseases (Abella et al. 2017; Cava 2017). It was revealed that leptin could increase the production of Th17 cytokines, which was related to the severity of asthma (Vollmer et al. 2022). Studies have shown that obesity-related asthma is more prevalent in women than in men, and the correlation between leptin and asthma is stronger in women than in men (Sood et al. 2006). Female asthmatic patients are more likely to have increased numbers of neutrophils in induced sputum (Telenga et al. 2012). To our knowledge, few studies have evaluated immune responses in the lungs of female mouse models with concomitant diet‐induced obesity and LPS/OVA + OVA-induced neutrophilic airway inflammation in females. Here, female mice were used to establish a neutrophilic airway inflammation mouse model. Our research aimed to investigate the role of leptin/obR signaling in a female obesity-related neutrophilic airway inflammation murine model to provide possibilities for the development of drugs for obesity-related asthma.

Intriguingly, our results indicated that serum leptin levels were increased in female obese mice compared with female lean mice. Previous studies indicated that OVA-sensitized and challenged obese mice showed higher serum levels of leptin (Shore et al. 2005; Han et al. 2017). Reportedly, inflammatory cytokines, including IL-1β and TNF-α, can induce leptin secretion from adipocytes (Grunfeld et al. 1996). Consistent with previous studies, our results showed that serum leptin levels were significantly upregulated after LPS/OVA + OVA treatment, suggesting that inflammatory responses may upregulate leptin secretion. Moreover, our results showed that in female obese mice with neutrophilic airway inflammation had more severe neutrophilic airway inflammation and higher levels of Th1/Th17 cytokines than female lean mice with neutrophilic airway inflammation. Previous studies have shown that leptin interacting with obR leads to inflammatory and immune responses in various immune diseases (Sánchez-Margalet et al. 2002; Cordero-Barreal et al. 2021). It was reported that phosphorylation of obR-b is crucial for obR activation and the initiation of leptin-mediated downstream signaling (Björnholm et al. 2007). We found that increased obesity-related neutrophilic airway inflammation was associated with high phosphorylation levels of obR-b.

Macrophages account for approximately 70% of lung immune cells and play an important role in various chronic lung diseases (Cai et al. 2014). Obesity-related inflammation has been suggested to be accompanied by an increase in M1 macrophage infiltration in adipose tissues (Bantulà et al. 2021). The majority of effector macrophages in nonallergic asthma were M1 macrophages, while the majority of macrophages in allergic asthma were M2 macrophages (Saradna et al. 2018). Moreover, M1 macrophages have been suggested to be associated with asthma severity (Oriss et al. 2017. Therefore, M1-phenotype macrophages can be used to evaluate obesity-related neutrophilic airway inflammation. In the current study, female obese mice with neutrophilic airway inflammation had a higher level of M1-polarized macrophages than female lean mice with neutrophilic airway inflammation. These results confirmed that proinflammatory M1 macrophage polarization is enhanced in obesity-related neutrophilic airway inflammation. Thus, molecular intervention to regulate M1 macrophages has the potential to treat obesity-related neutrophilic airway inflammation.

The effects of leptin on immune cell activation have been reported in various immune diseases (Kim et al. 2019). A previous study indicated that exogenous infusion of leptin could aggravate periodontitis by activating macrophages (Han et al. 2022). Another study indicated that hyperleptinemia induced by diet-induced obesity could increase the inflammatory response of macrophages (Monteiro et al. 2022). In the current study, we observed that M1-like phenotype macrophages were predominant in obR^+^ cells from an obesity-related neutrophilic airway inflammation mouse model. However, the interaction between leptin/obR signaling and M1 macrophages in the obesity-related neutrophilic airway inflammation mouse model needs to be further explored. We found that the Allo-Aca (an obR inhibitor) or obR-b deficiency significantly ameliorated M1 macrophage polarization in a murine model of obesity-associated neutrophilic airway inflammation. Significantly, we revealed an indispensable role of leptin/obR signaling in regulating M1 macrophage polarization in obesity-associated neutrophilic airway inflammation.

The mechanisms by which leptin/obR signaling affects M1 macrophage polarization have not yet been fully elucidated. Interestingly, leptin synergized with LPS/IFN-γ to induce remarkable M1 macrophage polarization by elevating obR-b phosphorylation, which could be reversed by Allo-Aca. Leptin alone did not exhibit significant effects on inducing M1 macrophage polarization. Previous studies have suggested that phosphorylation of STAT3 is activated in the M1 phenotype macrophages (Shi et al. 2020; Li et al. 2021). Moreover, it has been demonstrated that the JNK and AKT signaling pathways are activated by various stimulators during the process of M1 macrophage polarization (Liu et al. 2020; Cui et al. 2020; Chen et al. 2020). Leptin was also reported to activate the STAT3, AKT, and JNK pathways in breast cancer and ovarian cancer (Ghasemi et al. 2018; Kim et al. 2017b). In addition, leptin was reported to promote immune cells in obese tissues to produce inflammatory cytokines via the JNK/AKT pathway (Engin 2017). To date, few studies have suggested a role of the JNK/STAT3/AKT signaling pathway in leptin/obR-induced M1 macrophage polarization. We found that the JNK/STAT3/AKT signaling pathway might be involved in the proinflammatory effects of leptin/obR signaling via M1 macrophage polarization. In our present study, we identified that leptin synergized with LPS/IFN-γ to significantly increase the phosphorylation levels of members of the JNK/STAT3/AKT pathways in BMDMs, which was further reversed by Allo-Aca. To further explore whether the function of leptin/obR signaling in M1 macrophage polarization was dependent on the downstream of JNK/STAT3/AKT pathways, BMDMs were stimulated with Stattic (STAT3 inhibitor), Sp600 (JNK inhibitor), or MK-2206 (AKT inhibitor). Specifically, Stattic, Sp600, and MK-2206 effectively decreased the promoting effect of leptin on M1 macrophage polarization. Furthermore, leptin did not significantly upregulate LPS/IFN-γ-induced M1 macrophage polarization in db/db mouse-derived BMDMs, indicating the indispensable role of obR-b in leptin-mediated M1 macrophage polarization. All the results support the theory that leptin/obR signaling enhances M1 macrophage polarization via upregulating the phosphorylation of JNK/STAT3/AKT in vitro.

Macrophages and neutrophils are critical effectors in immune responses and inflammatory diseases (Siouti and Andreakos 2019; Mohr et al. 1981). Here, we demonstrated that the leptin/obR axis increased neutrophil infiltration in the airway, contributing to neutrophilic airway inflammation in an obese mouse model. CXCL2 is a chemokine secreted by macrophages that can promote neutrophil migration (Filippo et al. 2013; Zhang et al. 2019). In this study, we demonstrated that the leptin/obR axis signaling caused M1 macrophages to promote CXCL2 production through the JNK/STAT3/AKT pathways, which further increased neutrophil recruitment. Thus, we revealed that the leptin/obR axis play an important role in modulating neutrophil recruitment by activating M1 macrophages.

The pathogenic effects of leptin and M1 macrophages can be verified in asthmatic patients. Obese asthmatic patients with higher serum leptin levels and BMI are more likely to have severe clinical symptoms than nonobese asthmatic patients (Nadif et al. 2020; Chang et al. 2022; Zhang et al. 2017). Patients with obesity-related asthma are mostly characterized by low serum IgE levels and low numbers of eosinophils in sputum (Telenga et al. 2012; Gibeon et al. 2013). An increasing number of studies have shown that obesity-related asthma is related to neutrophil airway inflammation (Scott et al. 2011; Telenga et al. 2012). Consistent with the above studies, our study revealed that patients with obese asthma had significantly higher numbers of neutrophils in induced sputum and a higher level of serum leptin than patients with nonobese asthma. M1 macrophage-associated cytokines (IL-6 and TNF-α) were elevated in the sputum of patients with neutrophilic asthma (Shi et al. 2022). Leptin was found to participate in the inflammatory response by targeting macrophages (Mancuso et al. 2004; Raso et al. 2002). In our study, patients with obesity-related asthma had higher levels of M1 macrophages in induced sputum than patients with nonobese asthma. Moreover, the number of M1 macrophages in induced sputum was positively correlated with the level of serum leptin. The stratified analyses of serum leptin levels and the number of M1 macrophages between female and male patients have not displayed significant results (Additional file 4a–f). Collectively, these results suggest that leptin may be involved in neutrophil airway inflammation in obesity-associated asthma by affecting M1 macrophages, providing a novel direction for studying the pathogenesis of obesity-related asthma.

## Conclusions

In conclusion, we have demonstrated that leptin synergizes with LPS/IFN-γ to activate obR, thus promoting M1 macrophage polarization by activating the JNK/STAT3/AKT signaling pathways. Leptin/obR signaling promotes secretion of the neutrophil chemokine CXCL2 by M1 macrophages through the JNK/STAT3/AKT pathways, thereby promoting the chemotaxis of neutrophils. The leptin/obR axis participates in the pathogenesis of obesity-related neutrophilic airway inflammation in females by promoting M1 macrophage polarization, suggesting that targeting leptin/obR signaling may be an attractive strategy for treating neutrophilic airway inflammation in women with obesity-related asthma.

## Supplementary Information

**Additional file 1. Table S1.** The primary antibodies used in Western blotting.

**Additional file 2. Table S2.** A list of the antibodies used in flow cytometry.

**Additional file 3. Table S3.** The primers for each target gene used in RT-PCR.

**Additional file 4.** Stratified analyses between male and female patients. **a** The serum leptin levels between male and female patients. **b** Bar graph showing the number of M1 macrophages (CD11c^+^CD86^+^) between male and female patients. **c** The serum leptin level between male and female patients of NOA subjects. **d** The serum leptin level between male and female patients of OA subjects. **e** Bar graph showing the number of M1 macrophages (CD11c^+^CD86^+^) between male and female patients of NOA subjects. **f** Bar graph showing the number of M1 macrophages (CD11c^+^CD86^+^) between male and female patients of OA subjects. Data are expressed as the means ± SD.

**Additional file 5.** Plasma insulin level in mice (n = 5). Data are expressed as the means ± SD. *P < 0.05.

**Additional file 6.** **a** The weight change before or after LPS/OVA+OVA treatment in obese mice (n = 6). **b** The weight change before or after LPS/OVA+OVA treatment in lean mice (n=6). Data are expressed as the means ± SD.

## Data Availability

The datasets used and/or analyzed during the current study are available from the corresponding author on reasonable request.

## Abbreviations

BALF: Bronchoalveolar lavage fluid
BMDM: Bone marrow-derived macrophage
ACT: Asthma control test
FEV_1_: Forced exhaled volume at 1 s
FVC: Forced vital capacity
WBC: Blood white blood cell
ELISA: Enzyme-linked immunosorbent assay
iNOS: Inducible nitric oxide synthase
M-CSF: Macrophage colony-stimulating factor
H&E: Hematoxylin and eosin
PAS: Periodic acid–Schiff
MFI: Mean fluorescence intensity
STAT3: Signal transducer and activator of transcription 3
JNK: C-Jun N-terminal kinase
AKT: Protein kinase B
PBS: Physiological saline solution
BMI: Body mass index
