# Supplementary figures and images for: Leptin/obR signaling exacerbates obesity-related neutrophilic airway inflammation through inflammatory M1 macrophages
Source: Mol Med. 2023 Jul 24;29:100. doi: 10.1186/s10020-023-00702-w (PMC10367413; doi:10.1186/s10020-023-00702-w)

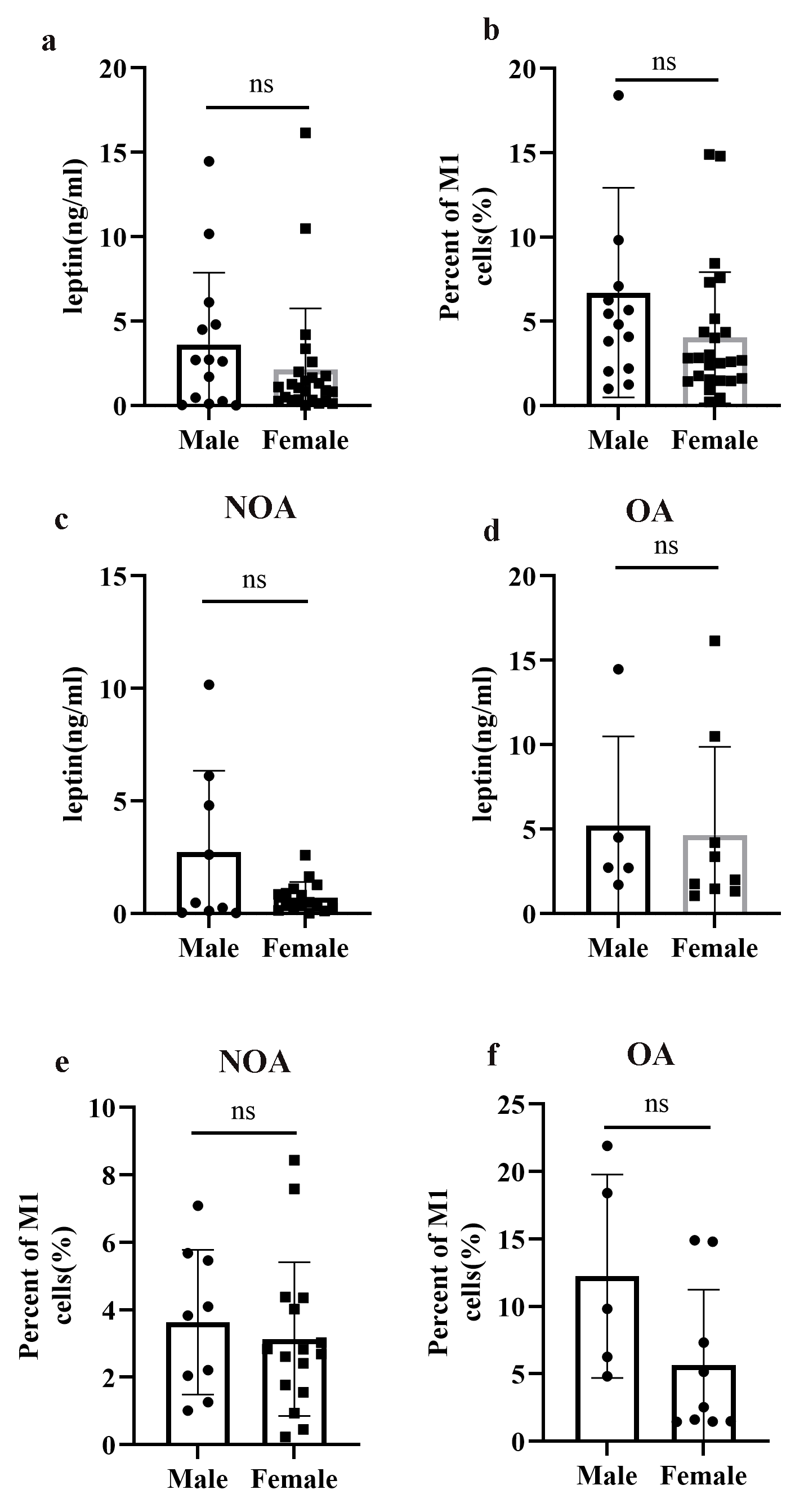

Supplement: Supplementary file 4 — Additional file 4. Stratified analyses between male and female patients. a The serum leptin levels between male and female patients. b Bar graph showing the number of M1 macrophages (CD11c+CD86+) between male and female patients. c The serum leptin level between male and female patients of NOA subjects. d The serum leptin level between male and female patients of OA subjects. e Bar graph showing the number of M1 macrophages (CD11c+CD86+) between male and female patients of NOA subjects. f Bar graph showing the number of M1 macrophages (CD11c+CD86+) between male and female patients of OA subjects. Data are expressed as the means ± SD. [file 10020_2023_702_MOESM4_ESM.tif]

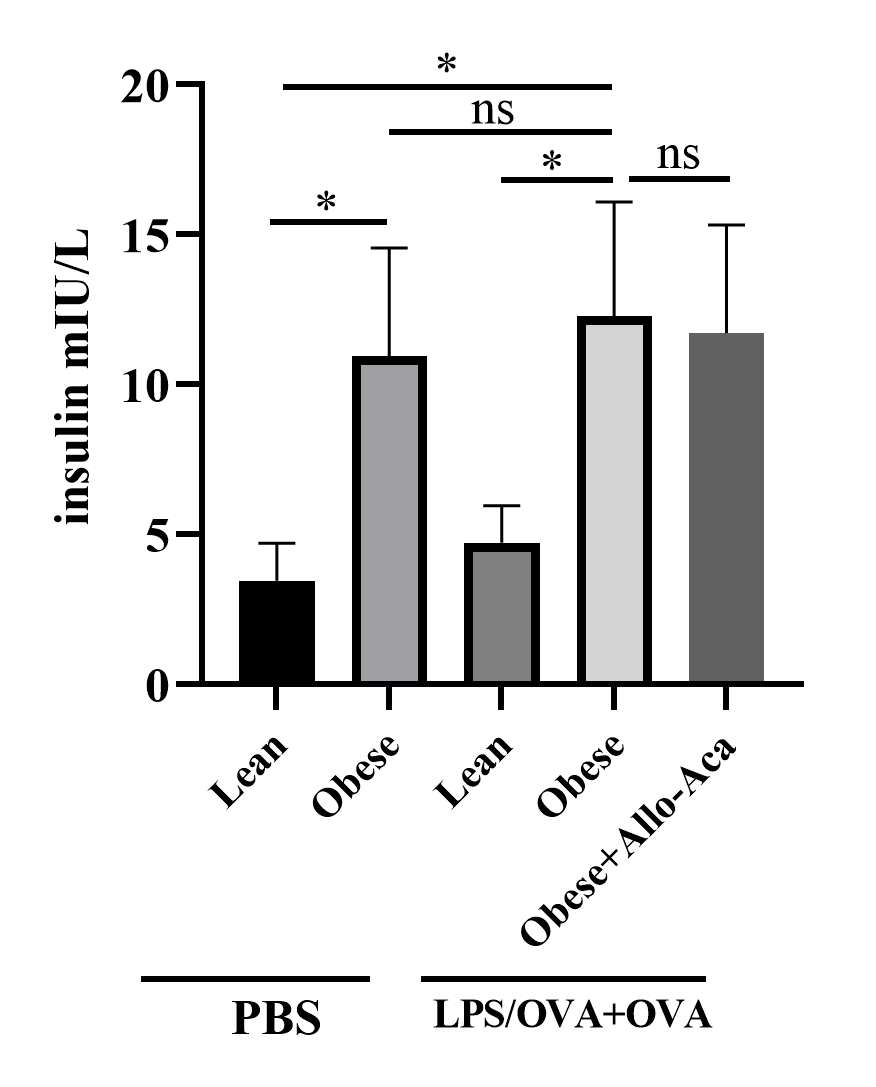

Supplement: Supplementary file 5 — Additional file 5. Plasma insulin level in mice (n = 5). Data are expressed as the means ± SD. *P < 0.05. [file 10020_2023_702_MOESM5_ESM.tif]

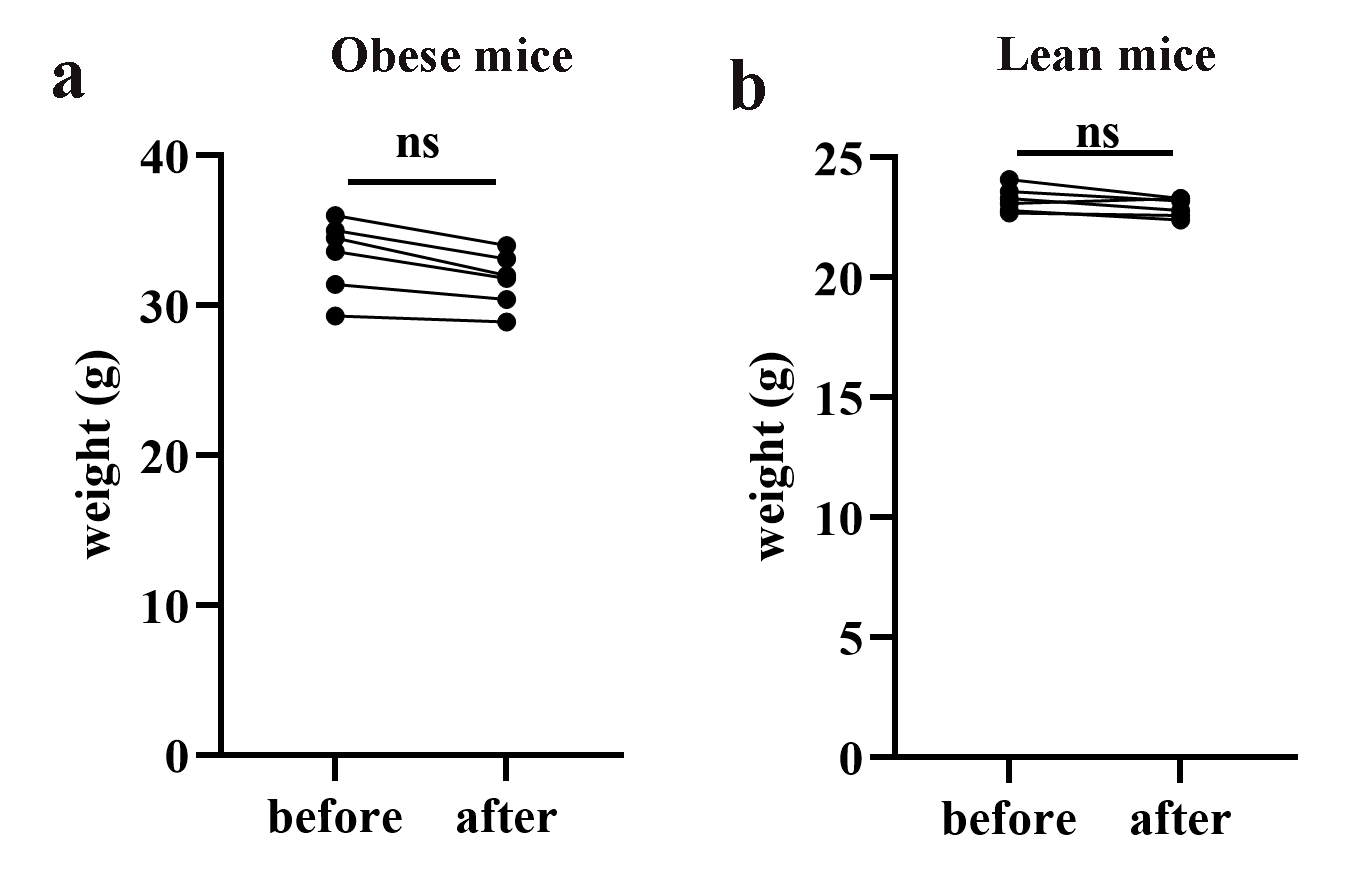

Supplement: Supplementary file 6 — Additional file 6. a The weight change before or after LPS/OVA+OVA treatment in obese mice (n = 6). b The weight change before or after LPS/OVA+OVA treatment in lean mice (n=6). Data are expressed as the means ± SD. [file 10020_2023_702_MOESM6_ESM.tif]
